# Quantum chemical calculations predict biological function: the case of T cell receptor interaction with a peptide/MHC class I

**DOI:** 10.3389/fchem.2015.00009

**Published:** 2015-02-10

**Authors:** Georgios S. E. Antipas, Anastasios E. Germenis

**Affiliations:** ^1^Division of Materials Technology, National Technical University of AthensAthens, Greece; ^2^Department of Immunology and Histocompatibility, School of Medicine, University of ThessalyBiopolis, Larissa, Greece

**Keywords:** pMHC-TCR interaction, atomic pair correlation, short range order, cumulative coordination, functional avidity, structure-function relationship

## Abstract

A combination of atomic correlation statistics and quantum chemical calculations are shown to predict biological function. In the present study, various antigenic peptide-Major Histocompatibility Complex (pMHC) ligands with near-identical stereochemistries, in complexation with the same T cell receptor (TCR), were found to consistently induce distinctly different quantum chemical behavior, directly dependent on the peptide's electron spin density and intrinsically expressed by the protonation state of the peptide's N-terminus. Furthermore, the cumulative coordination difference of any variant in respect to the native peptide was found to accurately reflect peptide biological function and immerges as the physical observable which is directly related to the immunological end-effect of pMHC-TCR interaction.

## Introduction

The intricate protein-protein interaction between an antigenic peptide-MHC complex and a T cell receptor (TCR) is of prominent physiological importance as it is a core aspect of the most crucial biological function: that of self-nonself discrimination. Clonal Selection Theory, the conceptual basis of contemporary Immunology initially put forth by MacFarlane Burnet in the mid 1950s (Burnet, 1959), proposed that discrimination between self vs. non-self requires a given T cell to be highly specific to a single cognate pMHC ligand (Cohn et al., 2007). Over time, however, accumulating experimental and theoretical evidence has grown in support of an opposite immunological theme: that effective immunity requires each TCR to be capable of interacting with a large number of potential antigenic peptides, a concept currently recognized as TCR degeneracy (Cohn, 2008). The premise of TCR degeneracy is characterized by varied behaviors of the cognate in respect to variant ligands. The cognate (natural) peptide is able to elicit a T cell's full program of activation events including proliferation and acquisition of effector functions. On the other hand, even single aminoacid substitutions in the sequence of the cognate ligand (yielding altered peptide ligands) may generate an ensemble of different outcomes, ranging from full lymphocyte activation to induction of early activation or even the very inhibition of the T cell toward antigenic stimulation. Based on their effects on T cell activation and differentiation, these altered peptides are classified as partial (weak) agonists and antagonists (Donermeyer et al., 2006).

Generation of different responses emanating from TCR binding with different altered peptide ligands is based on a molecular mechanism which is currently unclear. Theoretical approaches attempting to address the binding strength between pMHC and TCR, such as binding pocket energetics (Agudelo and Patarroyo, 2010), molecular dynamics (Wan et al., 2008) and thermodynamical modeling (Wan et al., 2005), invariably address the province of the first coordination shell of interatomic interactions in the pMHC-TCR complex. However, the similarity between agonist and antagonist pMHC structures suggests that engagement of MHC cleft sites by peptide residues is always via similar bond lengths, loosely reflecting similar bond orders (Bader and Laidig, 1991), such that we could argue that coordination degeneracy may well be reflected in non-meaningful contributions of the peptide toward pMHC binding energy.

A large number of MHC class I–restricted epitopes derived from the Tax protein have been both identified and exhaustively studied on the functional level. Tax protein is a transcriptional regulatory protein of the human T-cell leukemia virus type 1 (HTLV-1) playing a critical role in HTLV-1-associated leukemogenesis (Kannagi et al., 1991; Elovaara et al., 1993; Pique et al., 2000). It, thus, represents an attractive target for anti-cancer vaccine development (Sundaram et al., 2003). Here, we present a comparative atomistic study of the cognate Tax peptide and its variants; although these peptides present spectacular functional differences they exhibit near-identical stereochemistry (Ding et al., 1999). In the current study, we establish that even near-identical stereochemistry may consistently induce distinctly different quantum chemical behavior which, most importantly, is directly correlated to the immunological end-effect of pMHC-TCR interaction. Along these lines, we provide theoretical evidence that the immunological behavior of pMHC-TCR complexes is directly dependent on electron spin density and intrinsically expressed by the protonation state of the peptide's N-terminus. A likely eventuality of these findings is the inference of pMHC-TCR immunological behavior directly from peptide primary structure.

## Results

### Unprotonated peptide structures

**Figure 1:**
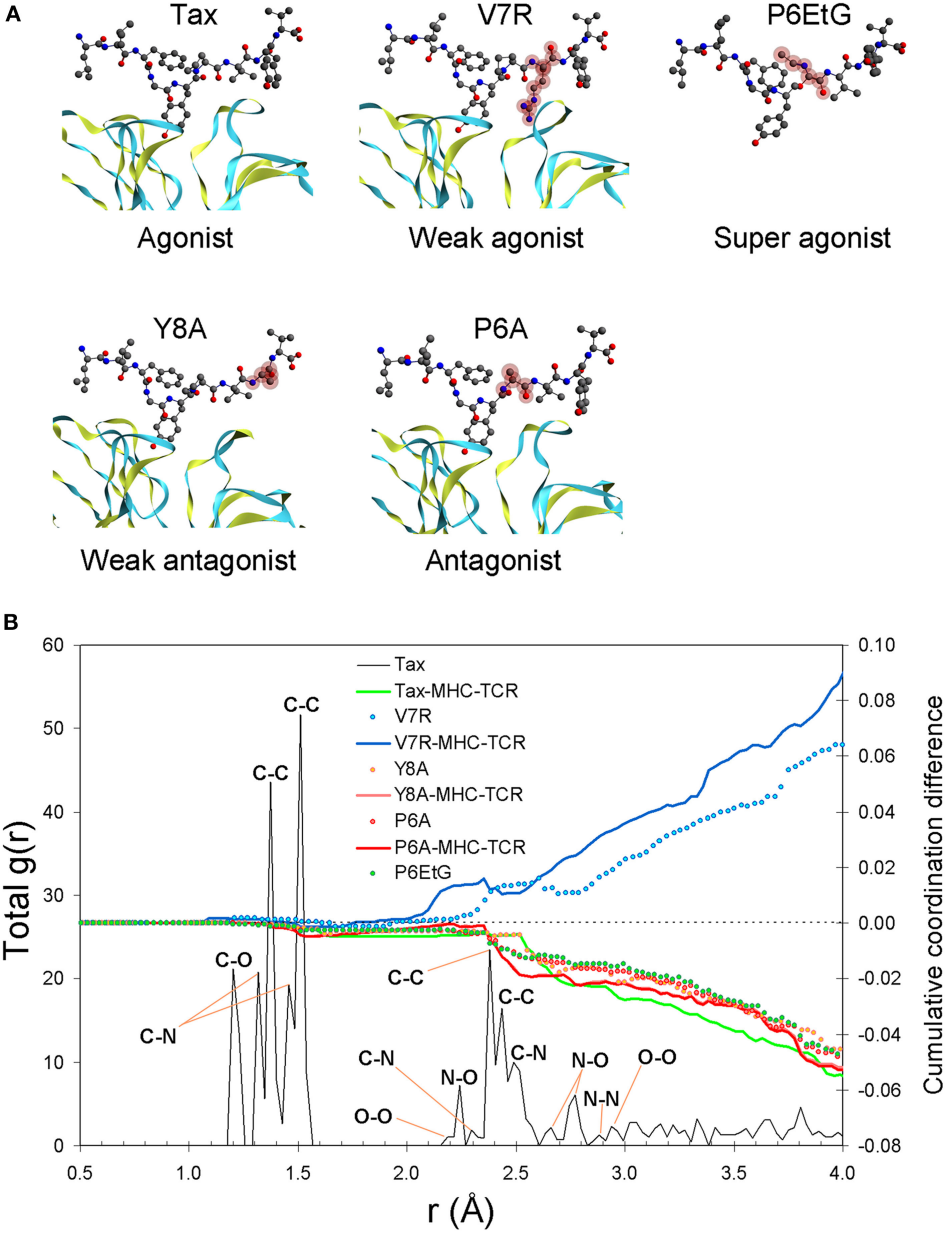
**Peptide unprotonated structure**. **(A)** The crystallized tertiary structures of Tax (agonist), V7R (weak agonist), Y8A (weak antagonist), and P6A (agonist). The TCR alpha and beta chains (shown as ribbons) are located underneath the peptides while the MHC alpha chain (not shown) is located over the peptide and encapsulates its hydrophobic portion. In all peptides, hydroxyl groups attached to the phenyl side chain of the residue in position five point toward the alpha chain of the TCR. Also shown is the P6EtG structure as constructed *in silico* in the current work (for details see the Methods Section). Atom color notation is C, gray; N, blue; and O, red and is followed throughout. Peptide substitutions in respect to Tax are highlighted in purple. **(B)** PDF of the Tax backbone and coordination differences in respect to Tax for the variants as well as for the as-crystallized pMHC-TCR complexes. All coordination deviations from the Tax structure are expressed as cumulative difference fractions.

Our results show that the pair distribution function (PDF) of the index Tax peptide, up to an interatomic distance of approximately 1.9 Å, is underpinned by carbon (C) nearest-neighbor partials, comprising the tertiary structure's first coordination shell, the latter encapsulating the topology of all bonded atomic interactions. Interestingly, atomic structure within the first coordination shell yields near-identical correlation statistics for all peptides (henceforth, extensive similarity within the first coordination shell will be referred to as coordination degeneracy or, simply, degeneracy, in the context of structure). Such first coordination shell degeneracy is, in fact, characteristic of the entire pMHC-TCR complex as well as of the isolated pMHC (see Figure 1B). Beyond the limit of 1.9 Å and up to approximately 3.3 Å lies the second coordination shell of interatomic interactions, in which peptide atomic structure diverges. Within this region, and particularly after the C-C second nearest-neighbor peak at 2.9 Å, the V7R agonist variant exhibits systematic over-coordination in respect to the index peptide, while the P6EtG super agonist variant is under-coordinated along with all of the antagonist peptides.

### Protonated peptide structures

Protonation of the as-crystallized peptide backbones is accompanied by the possibility of spin polarization, the latter being naturally introduced as a fundamental parameter of the peptide ground state under a particular formal charge (see Table 1). The facility for spin polarization is also on a par with mounting evidence that biological complexes, such as π-conjugated zwitterionic molecules, may exhibit a ferromagnetic ground state (Shelton et al., 2011). In this study, polarization is regarded to be owing to a stimulus external to the peptide. Additionally, we cater for the eventuality of hydrogen abstraction from hydrophilic (phenol OH^−^) side chains (to be referred as triggering side chains, henceforth) upon contact with the TCR.

Table 1**PMHC-TCR complex and peptide DFT data**.

**Table:** 

**Model designation**	**Tax z0 s0 p**	**V7R z1 s0 p**	**P6EtG z0 s2 p**	**Y8A z0 s0 p**	**P6A z0 s0 p**
Peptide	Tax	V7R	P6EtG	Y8A	P6A
Immunological designation	Agonist	Weak agonist	Super agonist	Weak antagonist	Antagonist
Sequence (contributors to z in bold, variants in respect to Tax underlined)	**L**LFGYPVY**V**	**L**LFGYP**R**Y**V**	**L**LFGY EtG VY**V**	**L**LFGYPVA**V**	**L**LFGYAVY**V**
Formal charge	0	1	0	0	0
Spin polarization	0	0	2	0	0
N-C termini distance (Å)	24.699		24.308	24.972	24.477
Density (g/cm^3^)	0.281	0.287	0.299	0.247	0.273
Number density (atoms/Å^3^)	0.025	0.025	0.026	0.022	0.024
First coordination shell radius (Å)	1.856	1.829	1.827	1.888	1.769
Binding energy (ma.u)	−57.745	−56.983	−56.746	−57.888	−57.644
Zwitterion	Yes	Yes	Yes	No	No

**Table:** 

**Model designation**	**Tax z-2 s0 u**	**Tax z-2 s2 u**	**V7R z-1 s1 u**	**P6EtG z-2 s0 u**	**P6EtG z-2 s2 u**	**Y8A z-1 s0 u**	**P6A z-2 s0 u**	**P6A z-2 s2 u**
Peptide	Tax		V7R	P6EtG		Y8A	P6A	
Immunological designation	Agonist		Weak agonist	Super agonist		Weak antagonist	Antagonist	
Sequence (contributors to z in bold, variants in respect to Tax underlined)	**L**LFG**Y**PV**YV**		**L**LFG**Y**PR**YV**	**L**LFG**Y** EtG V**YV**		**L**LFG**Y**PVA**V**	**L**LFG**Y**AV**YV**	
Formal charge	−2		−1	−2		−1	−2	
Spin polarization	0	2	1	0	2	0	0	2
N-C termini distance (Å)	24.699		24.392	24.308		24.972	24.477	
Density (g/cm^3^)	0.281	0.281	0.290	0.299	0.299	0.248	0.260	0.266
Number density (atoms/Å^3^)	0.024	0.024	0.025	0.026	0.026	0.022	0.023	0.023
First coordination shell radius (Å)	1.769	1.769	1.829	1.798	1.769	1.792	1.769	1.827
Binding energy (ma.u)	−57.195	−57.308	−56.300	−56.485	−56.431	−57.750	−57.258	−57.027
Zwitterion	Yes	Yes	Yes	Yes	Yes	No	No	No

The notation of model designation is as follows: Peptide name followed by the letters “z” and “s” defining the peptide formal charge and spin polarization, respectively. Peptides with protonated hydrophilic (triggering) side chains are denoted by “p” and “f” while deprotonated side chains are symbolized by “u.” Notation is followed throughout. The binding energy has been normalized by the number of electrons in every peptide.

**Figure 2:**
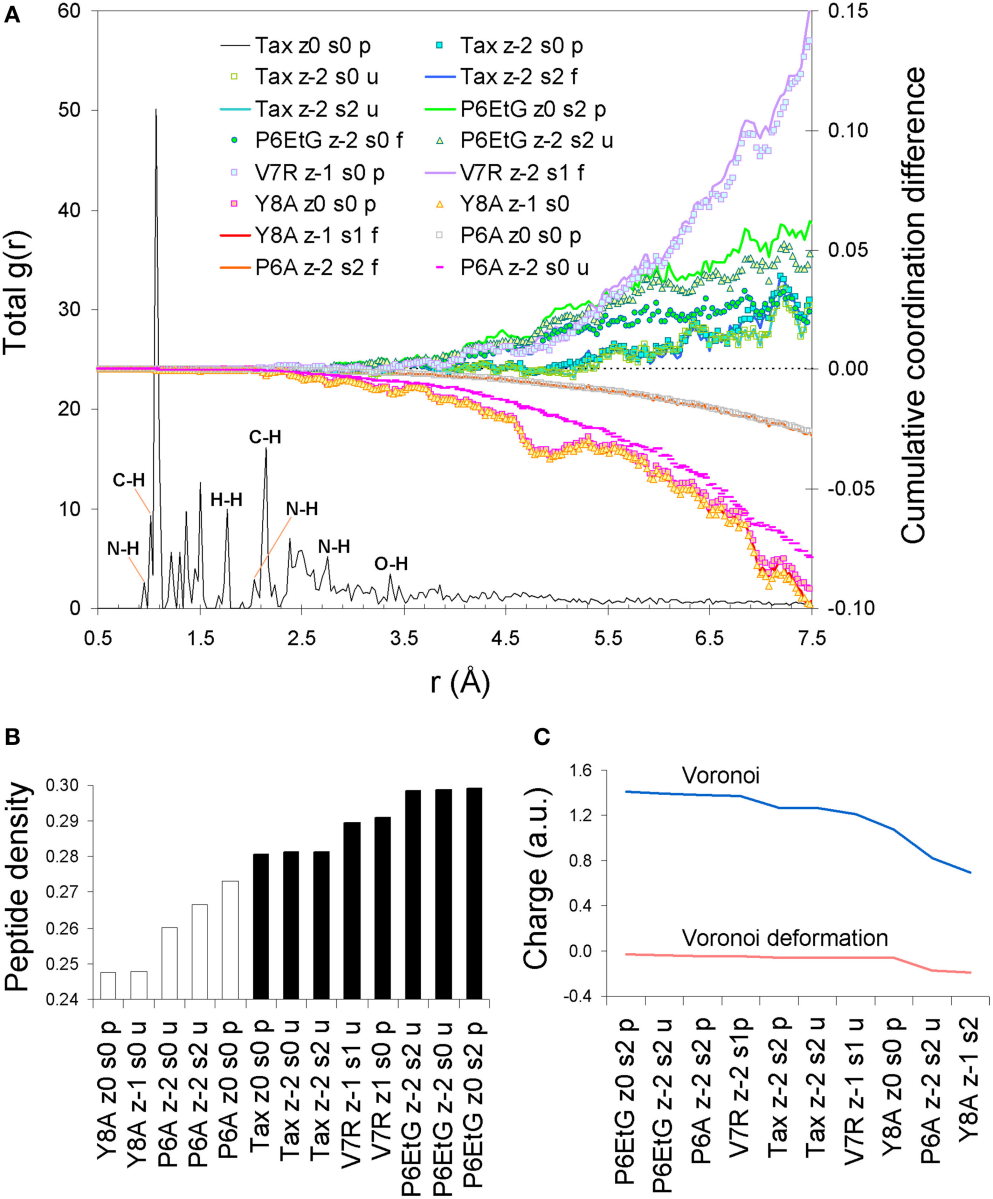
**Peptide protonated structure**. **(A)** PDF of the protonated Tax tertiary structure and cumulative coordination differences in respect to Tax for the Tax, V7R, Y8A P6A, and P6EtG variants for singlet and triplet states. **(B)** Average peptide density. **(C)** Voronoi charge and Voronoi deformation charge variation for a range of representative peptide states.

Upon protonation of the peptide backbones, the feature of degeneracy within the first coordination shell is maintained (see Figure 2A). However, beyond the first shell, protonation induces a bias which appears symptomatic to immunological function: all agonist peptides are systematically over-coordinated in respect to (index) Tax while antagonist variants are under-coordinated. Moreover, this coordination consistency is detected in all combinations of peptide formal charge and polarization. Interestingly, all agonists are also of a higher average density than antagonist peptides (see Figure 2B). One possible reason for the systematic over-coordination of the agonists may be the efficient expulsion of water molecules from the TCR cleft due to peptide increased density. This would also be in alignment with the entropic benefit occurring via expulsion of water molecules from the cleft during formation of the immune synapse (Schamel and Reth, 2007). Equally intriguing is the fact that variant peptide deviations from the average Tax stereochemistry become pronounced at a PDF length corresponding to the first N-N PDF peak of 2.87 Å. This indicates that any adjustments made by the peptide to accommodate its tertiary structure are made on an inter-residual scale rather than within residues and suggests increased peptide rigidity.

### The effect of spin polarization

**Figure 3:**
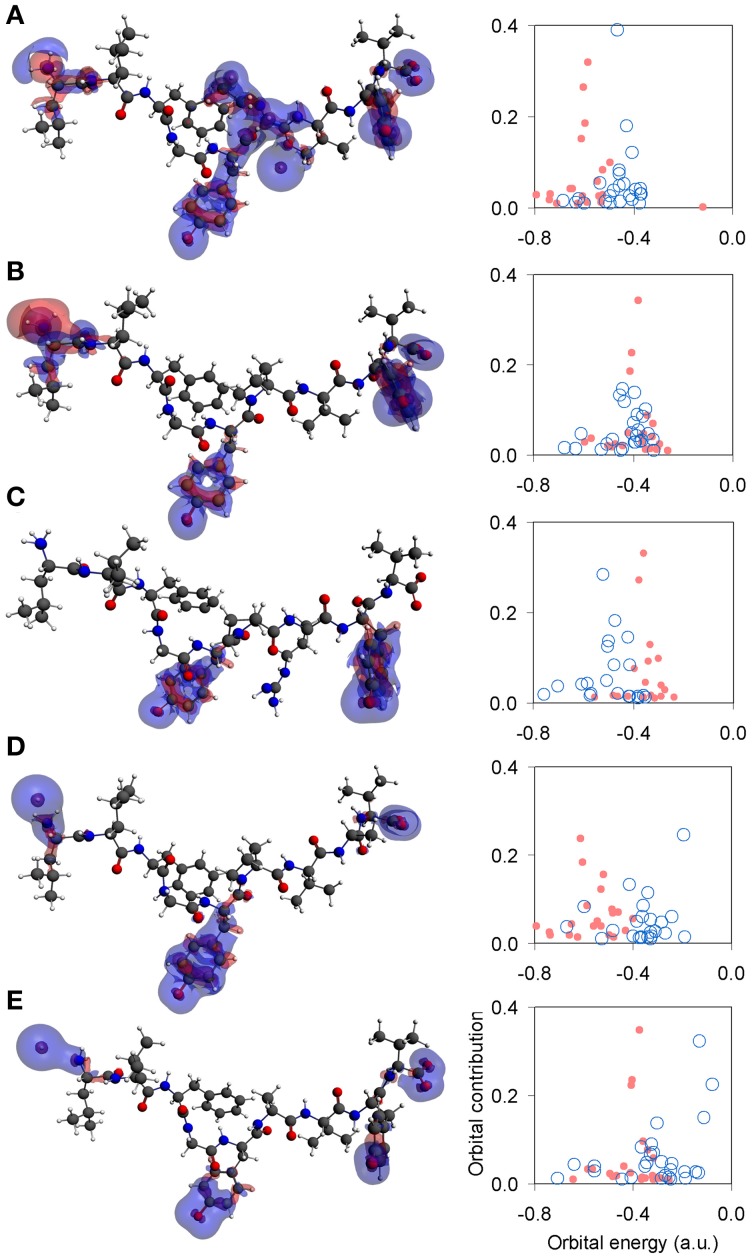
**Laplacian of the spin density and N-terminus state**. Topology of the Laplacian of the spin density (α–β spin) for representative tertiary structures under spin polarization. All iso-density surfaces are drawn at 0.001 a.u. Charge concentration and charge depletion are shown as red and blue surface segments, respectively. From **(A–E)**: P6EtG z-2 s2 u, Tax z-2 s2 u, V7R z-1 s1 u, Y8A z-2 s1 u, and P6A z-2 s2u. Next to each structure is a corresponding graph of the peptide's N-terminus orbital contribution (fraction), upon transition from the protonated (light red full circles) to the deprotonated (light blue open circles) state of its triggering side chains.

Unpolarized peptides, regardless of charge and immunological identity (agonist-antagonist), were always found to adopt a stable zwitterionic singlet state. Based on the Laplacian of the electron density, charge depletion mainly over peptide backbones, as well as charge concentration over side chains and, in particular, over polar hydrogens was also found to form a near-identical motif. However, the introduction of polarization causes formation of a stable N-terminus ammonium (NH^+^_3_) group exclusive to agonists, and an altogether lack of zwitterionic expression in the case of antagonists. Voronoi charge analysis reveals that the fundamental reason for such different behavior is electron localization over the peptide N-terminus, reflected on the charge of the terminus nitrogen (N) atom. Upon deprotonation of the triggering side chains, the flow of electron charge into the N-terminus nitrogen (as shown by the Voronoi deformation density, see Figure 2C) is negligible in the case of agonists while more substantial in the case of antagonists. Also, the Laplacian of the spin density reveals that the terminus ammonium (NH^+^_3_) group in agonists is surrounded by a shell of charge localization (high potential energy) wrapped in an outer surface of charge depletion (high electron kinetic energy). In contrast, charge is always depleted over the antagonist N-termini amine (NH_2_) groups (see Figure 3). The varied response of the N-terminus between agonist and antagonist peptides upon deprotonation of the triggering side chains is fundamentally reflected upon the manner in which the N-terminus nitrogen redistributes its molecular orbital contributions. Regardless of spin, formal charge and immunological identity, peptides present a tendency to maintain their t_1u_ contributions largely around −0.4 a.u., with varying success. All agonists accommodate deprotonation by shifting nitrogen orbital contributions to lower energies, while antagonists shift to higher energies. Also, super agonist P6EtG exhibits the lowest energy deviation in its orbital contributions, with the deviation steadily increasing for Tax and weak agonist V7R. The extent of energy deviation could be argued as loosely correlated to the strength of the immunological identity.

## Discussion

Although the ability of the pMHC complex to activate T cells is customarily addressed via the affinity (or t_1/2_) of peptide binding to the TCR, t_1/2_ operates on the molecular (phenotypic) level and is not physically relevant to the quantum state of the peptide. On the contrary, charge and spin polarization are both central to the peptide quantum state and, as previously mentioned, there is firm experimental evidence of the existence of both in various biomolecular polymers (Shelton et al., 2011); if, however, charge and spin are indeed allowed to act on the peptide, the fundamental question of which charge/spin combination might be probable under “normal” conditions is inherently raised. Fortunately, all peptide structures yield a very consistent cumulative coordination profile, which explicitly differentiates the agonist from the antagonist peptides, as shown in Figure 2A. Therefore, regardless of which particular charge/polarization pair might actually occur at normal conditions, cumulative over-coordination of the agonist vs. under-coordination of the antagonist peptides in respect to Tax is always observed. Furthermore, if the discussion is restricted to the crystallized peptide structures, both Figures 2A,B are suggestive of the same principle: cumulative over-coordination (higher density) in respect to the native peptide signifies a weaker agonist, while cumulative under-coordination (lower density) flags an antagonist peptide. Moreover, pronounced coordination deviations from the native peptide signify correspondingly weaker peptide avidity, as portrayed by the behavior of V7R and Y8A. Therefore, cumulative coordination difference in respect to the native peptide is a physical observable symptomatic of biological function, albeit on the provision that the stereochemistry of the native peptide is known. Most importantly, however, our results indicate that the protonation state of the N-terminus is a physical observable linked to peptide functionality, which may be employed to characterize avidity even when native peptide stereochemistry is unavailable. Furthermore, if the N terminus of agonist peptides were to be found to retain its protonation (ammonium) state during the engagement it would signify the presence of causality. One aspect required for the conservation of the protonation state is increased rigidity of the hydrophobic portion of the peptide (and/or increased rigidity of the MHC structure surrounding the peptide's hydrophobic portion). The degree of causality cast upon the protonation state of the N terminus could, for example, be approached by comparing our current results with N-termimus protonation states in crystallized pMHC complexes, in the absence of the TCR.

The implication of a spin-polarized peptide is of particular merit as it may relate to the ability of a TCR to recognize different peptides by virtue of conformationally-induced spin moments, rather than sheer topology-based affinity, a condition that would render the immune recognition as fundamentally spin-specific. In the premise of such selective spin delocalization, the quantum feature attributed to immunological identity is the strive for the preservation of peptide zwitterionic state upon deprotonation of the triggering side chains during pMHC engagement by the TCR. Based on the indication of increased peptide rigidity, we deem that the basis for efficient immune recognition is principally owing to the effect of pMHC structure. Therefore, the condition required for a protective immune response seems to be an inborn characteristic of the pMHC complexes, which is gained as they are formed during antigen processing and presentation of antigen peptides. Hence, agonism is in-bread into the peptide, already upon its presentation on the MHC. The substantial pMHC rigidity vs. TCR is currently supported in the literature (Gakamsky et al., 2004; Schamel and Reth, 2007), confirming our view. An additional implication precipitating from this approach is that fixed TCR specificity (i.e., one TCR/one pMHC) would not be required, as TCR specificity would be disengaged from cross-reactivity, a possibility which has already been raised experimentally (Wooldridge et al., 2012). Furthermore, if first coordination shell degeneracy is symptomatic of all protein structure, the current methodology may have applicability to any pMHC-TCR complex.

## Methods

### Peptides

The crystallized pMHC-TCR complexes considered were the cognate HTLV-1 Tax peptide (LLFGYPVYV, PDB entry 1AO7) (index peptide) along with its singly substituted variants V7R (LLFGYPRYV, PDB entry 1QSE), Y8A (LLFGYPVAV, PDB entry 1QSF), and P6A (LLFGYAVYV, PDB entry 1QRN) which have been reported as stereochemically similar whilst biologically diverse (Ding et al., 1999). All peptides are presented by HLA-A201 and bound to the human A6TCR. Based on cell assays, kinetic and thermodynamic measurements, the P6A and Y8A variants are a strong and a weak antagonist respectively, while V7R behaves as a weak agonist or null peptide (Ding et al., 1999). Moreover, the variant P6EtG (LLFGYEtGVYV) was also compared against the index peptide; P6EtG, characterized as a super agonist by kinetic and thermodynamic measurements (Baker et al., 2000), was reconstructed *in silico* from the protonated P6A tertiary structure by substitution of the A6 by an EtG residue and subsequent Density Functional Theory (DFT) relaxation of the EtG atoms along with peptide atoms within a radius of 4 Å of each of the EtG atom centers. The relaxation radius of 4 Å was chosen as the largest PDF distance beyond which no appreciable structural features exist (see Figure 1A for peptide stereochemistry).

We note that all PDB entries used represent crystallized structures of fully formed pMHC-TCR clefts for which the entropic benefit involved in the expulsion of water molecules is high (Schamel and Reth, 2007). In accordance to this view, our work assumes absence of water molecules and treats the peptide in the gas phase. However, we consider that even in the case water molecules were, in fact, retained in the cleft, their combined effect would, evidently, produce the crystallized structures at hand but water molecules would potentially influence the hydrophilic part of the peptide (i.e., the triggering side chains); we have taken this eventuality into account by considering various protonation possibilities (see Table 1).

### Calculation of pair correlation functions

The PDF, also symbolized as *g*(*r*), is a statistical representation of interatomic distances (Antipas et al., 2012). The PDF was calculated by initially constructing the histogram of interatomic distances in respect to the real space coordinate, *r*. Calculation of the histogram involved the initial partition of space into bins of finite width. The most appropriate bin size, Δ*r*, may generally be derived by experimentation; it is normally preferable to select the largest bin size for which fine PDF details are maintained. In the current work the most appropriate bin size was determined to be 0.1 Å, following a number of trials. Mathematically, the PDF was defined as

(1) $$g(r)=\frac{1}{2\pi Nr^{2}\rho_{0}}\sum_{j\;=\;1}^{N}\sum_{i\;>\;j}^{N}\delta (r-r_{ij})$$

where *N* is the number of peptide atoms, δ is the Kronecker delta function and ρ_0_ is the number density expressed as *N/V*, where *V* is the volume of the simulation box containing the peptide. On the basis of the PDF, the radial distribution function (RDF), symbolized as R(r), was then expressed as

(2) $$\text{R}(\text{r})=4\pi {\text{r}}^{2}\rho_{0}\text{g}(\text{r})$$

and it was employed to calculate of atomic coordination. In physical terms, coordination is the number of atoms, *n*^*r*_2_^_*r*_1__, within a spherical shell defined by radii r_1_ and r_2_, where r_1_ < r_2_, along the real space coordinate

(3) $${\text{n}}_{{\text{r}}_{1}}^{{\text{r}}_{2}}=\int_{{\text{r}}_{1}}^{{\text{r}}_{2}}\text{R}(\text{r})\text{dr}=4\pi \rho_{0}\int_{{\text{r}}_{1}}^{{\text{r}}_{2}}\text{g}(\text{r}){\text{r}}^{2}\text{dr}$$

The latter quantity n expresses the average coordination between r_1_ and r_2_, to be referred to simply as coordination. Consequently, the cumulative coordination for each peptide up to any value of interatomic distance r_2_ was defined as the integral of Equation (3) by setting r_1_ equal to zero. Moreover, we calculated the running difference between any pair of such cumulative coordination integrals. Coordination as defined by Equation (3) did not take into account the species of atoms and its PDF is, hence, referred to as the total PDF. The additional constrain of the atomic species on Equation (3) yielded the atomic partial coordination, for which cumulative coordination differences may also be calculated as laid out previously. In the current work, the first coordination shell of peptide tertiary structure was defined as the real space coordinate limit which encapsulated the first peak of the total PDF and its physical significance is that it is shaped by all bonded interactions, regardless of their respective placement within the structure.

### Quantum chemical calculations

Spin unrestricted, all-electron DFT calculations were performed with the Amsterdam density functional (ADF) program (Fonseca Guerra et al., 1998, 2004; te Velde et al., 2001; SCM, 2010) within the realm of the generalized gradient approximation (GGA). Electron exchange and correlation were addressed by the BLYP (Becke, 1988; Lee et al., 1988) functional. Single-electron wavefunctions were expanded using the TZ2P uncontracted Slater-type orbital (STO) basis set, (a triple-ζ basis set with two sets of polarization functions) for all atoms. Relaxation simulations were followed by single point calculations for all structures to ensure full SCF convergence; however, non-aufbau occupations did occur and these simulations were discarded.

## Author contributions

Both authors contributed equally to this work. Georgios S. E. Antipas assembled the data and performed the DFT modeling. Georgios S. E. Antipas and Anastasios E. Germenis wrote the main paper. Both authors discussed the results and commented on the manuscript at all stages.

### Conflict of interest statement

The authors declare that the research was conducted in the absence of any commercial or financial relationships that could be construed as a potential conflict of interest.
